# Correction to: Poor sensitivity of "AccuPower SARS‑CoV‑2 real time RT‑PCR kit (Bioneer, South Korea)"

**DOI:** 10.1186/s12985-021-01506-2

**Published:** 2021-02-18

**Authors:** Byron Freire‑Paspuel, Miguel Angel Garcia‑Bereguiain

**Affiliations:** One Health Research Group, Universidad de Las Américas, Quito, Ecuador

## Correction to: Virol J (2020) 17:178 https://doi.org/10.1186/s12985-020-01445-4

After publication of our article [1] it was brought to our attention that we had not included sufficient details of the extraction method. All the samples were processed with the same RNA extraction kit "AccuPrep Viral RNA extraction kit IVD" (Bioneer, South Korea), and the same RNA extraction was used for both RT-PCR protocols. A maximum of a cycle of – 80 °C frozen/thawed was applied for some samples that could not be processed with the Bioneer RT-PCR kit within the same day that the CDC protocol. We have also tested in our lab that RNase P values do not vary after a single cycle of frozen/thawed as we do not observe an increase in RNase P Ct values when a "inconclusive" sample has to be repeated. So, we can certainly assure that no RNA degradation happens for the experimental conditions described in our study.

In addition, our Competing Interests statement was not correct. It should read "The authors declare that their affiliation institution "Universidad de Las Américas" is involved in producing a SARS-CoV-2 RT-PCR kit. However, this kit was not used in the present study."
